# Worldwide food recall patterns over an eleven month period: A country perspective

**DOI:** 10.1186/1471-2458-8-308

**Published:** 2008-09-10

**Authors:** Tamás Nepusz, Andrea Petróczi, Declan P Naughton

**Affiliations:** 1 Budapest University of Technology and Economics, Department of Measurement and Information Systems, H-1521 Budapest, P.O. Box 91, Hungary; 2 School of Life Sciences, Kingston University, Penrhyn Road, Kingston, London, KT1 2EE, UK

## Abstract

**Background:**

Following the World Health Organization Forum in November 2007, the Beijing Declaration recognized the importance of food safety along with the rights of all individuals to a safe and adequate diet. The aim of this study is to retrospectively analyze the patterns in food alert and recall by countries to identify the principal hazard generators and gatekeepers of food safety in the eleven months leading up to the Declaration.

**Methods:**

The food recall data set was collected by the Laboratory of the Government Chemist (LGC, UK) over the period from January to November 2007. Statistics were computed with the focus reporting patterns by the 117 countries. The complexity of the recorded interrelations was depicted as a network constructed from structural properties contained in the data. The analysed network properties included degrees, weighted degrees, modularity and *k*-core decomposition. Network analyses of the reports, based on 'country making report' (*detector*) and 'country reported on' (*transgressor*), revealed that the network is organized around a dominant core.

**Results:**

Ten countries were reported for sixty per cent of all faulty products marketed, with the top 5 countries having received between 100 to 281 reports. Further analysis of the dominant core revealed that out of the top five transgressors three made no reports (in the order China > Turkey > Iran). The top ten detectors account for three quarters of reports with three > 300 (Italy: 406, Germany: 340, United Kingdom: 322).

**Conclusion:**

Of the 117 countries studied, the vast majority of food reports are made by 10 countries, with EU countries predominating. The majority of the faulty foodstuffs originate in ten countries with four major producers making no reports. This pattern is very distant from that proposed by the Beijing Declaration which urges all countries to take responsibility for the provision of safe and adequate diets for their nationals.

## Background

The worldwide importance of a safe diet is reflected in the in depth discussions and reports leading up to and following the Beijing Declaration on Food Safety [1-11]. Following the World Health Organization Forum in November 2007, the Declaration was adopted by over 50 countries and identified the right of the individual to a safe and adequate diet along with guidance to countries for its provision. The Declaration stipulates the essential public health function of food safety controls and, moreover, the application of equal food safety measures within and between countries. It urges all countries to "establish food safety authorities ...within a comprehensive production-to-consumption legislative framework".

A great deal of discussion has occurred regarding the development of a new concerted model for food safety controls in Europe. Following decades of piecemeal regulation, the European Food Safety Authority (EFSA) was established in 2002 to provide a platform for scientific advice and the commitment required to ensure customer protection [7]. The final format veered from the US Food and Drug Administration approach, which has a focus on the three strands of risk-assessment, -management and -communication. In contrast, the EU model has separated risk assessment from management to ensure that "the control must be at the heart of the Commission's risk management process".

In 1979, the European Commission set up the Rapid Alert System for Food and Feed (RASFF), a key instrument for customer protection [12]. Although, it has the principal aim of alerting countries to immediate hazards, the RASFF provides a useful database for studying historical trends in food safety issues, along with the potential to predict future risks [13]. Whatever the nature of the potential hazard, be it a chemical, microbiological, parasite, packaging or contaminant of human origin, the Beijing Declaration fully recognizes the need for improved and continued annual reporting systems. A key aspect of the Declaration is that each country should be actively engaged in the process. Numerous reports exist on the breakdown of food types, hazard types and countries of origin and reporting body [12,13]. However, despite the Beijing Declaration, there is a paucity of reported studies of analyses of patterns from a nation perspective – both the reporting countries and countries of origin for hazardous foods.

The aim of this study is to retrospectively analyze the patterns in risk reporting within and between countries to identify the principal hazard generators and gatekeepers of food safety. The reporting period has been chosen as the eleven months prior to the Beijing Declaration with a view to a future comparative study being possible to monitor enhanced or reduced adherence to the Declaration over time.

## Methods

The food recall data set was collected by the LGC (UK) over the period from January to November 2007, inclusive. The data set contained detailed information which included: Date; Agency; Alert Reference; Type of Alert; Country Notified by; Company; Reasons for recall; Category; Coded category; Metal categories; Country of Origin; Type of Control; and Status. Descriptive statistics and statistical analyses were computed by using Excel, SPSS 15.0 and the R statistical framework [14]. Network properties such as degrees, weighted degrees, modularity and k-core decomposition were performed and graph visualisations were drawn by using the igraph library for network science [15]. Country codes with full country names are listed in Additional file 1: (Weighted and unweighted *in-degree *(transgressor) and *out-degree *(detector) for all countries). A brief explanation and calculation of modularity is provided in Additional file 2: (Definition of modularity).

## Results and discussion

### Network analyses

With the rapid improvement in computational power, network analysis has become a useful tool to analyze complex information for underlying structure or patterns, otherwise undetectable with descriptive analyses [16]. Information on the network size, connections, and structural properties such as the number of layers or cluster formations is capable of revealing important collective information about the *system *[17]. Mathematical manipulations utilised in this paper can be equally applied to data containing hundreds (such as the present data), thousands or millions of data points (nodes) which can be visualised by graphical methods. Owing to the recently discovered similarities in structural properties among different systems, ranging from social groups through scientific collaboration, traffic, and neural networks to metabolic networks and food webs, describing these systems by their network properties has turned to be highly instrumental [18]. The deconvolution approach to complex interrelationships using network analyses has been applied to diverse systems including i) drug-therapy interactions, ii) evolution-ecology investigations, and iii) elucidating mechanisms of disease aetiology [19-22].

**Figure 1:**
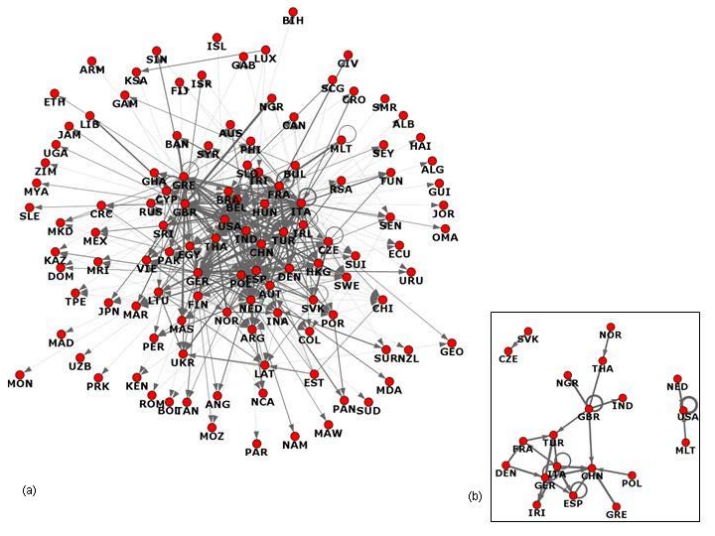
Graph representation of countries involved in the food alert/recall between January and November, 2007; (a) full graph; (b) subgraph with degrees > M ± SD.

The complexity of the recorded interrelations for the food recall data is best to be pictured as a network, which can be constructed from structural properties (relationships) contained in the data. Figure 1a depicts all 117 countries involved in food alerts either as detector or transgressor (or both), connected by 574 links. Two countries are linked if one of them reported on the other at least once during the examined time period where the direction of the arrow reflects the roles of the countries: arrows originate from detectors and are oriented towards transgressors. Mutual arrows are present between two countries if they both reported on each other. The weight assigned to an arrow reflects the number of reports with the width of the arrow proportional to the logarithm of its weight. Thus, the role of a country in the network is reflected by the number, direction and width of its adjacent arrows.

The average number of reports between countries (i.e. the average edge weight) was 4.28 ± 9.11 (maximum weight = 126) and only 29 (5.1%) links had weights above the mean plus one standard deviation (Figure 1b). These links will be called strong links from now on. It is notable that the distribution of the weights was positively skewed with 456 links below the mean. This suggests that the full graph is predominantly connected by relatively weak links. The weight distribution is further discussed below in the 'breakdown of reporting pattern' section. Interestingly, the largest distance in the network (the so-called diameter of the network) is only 5, even though there are a lot more countries in the dataset.

Countries connected with strong links (Fig 1b) were: TUR, ITA, GER, FRA, USA, MLT, NED, NOR, THA, NGR, IND, GBR, CHN, GRE, POL, ESP, IRJ, DEN, CZE, SVK. Of these 20 countries, the UK, ESP and GER made alerts/reports on products from their own countries (as indicated by the 'loops' in Fig 1b). For clarity, the UK is labeled as GBR. To unveil the inner structure of the network, attempts were made to decompose it to several smaller clusters (densely connected subgroups that are connected by a small number of edges). A common approach to decompose a network is to find a partition of the nodes that maximizes the measure called modularity [23]. (For a more detailed description of modularity, refer to Additional file 2: Definition of modularity). Modularity maximization was performed by the heuristic introduced by Latapy and Pons [24]. As a rule of thumb, Newman stated that a modularity value < 0.3 indicates the absence of a strong clustered structure [23]. The best modularity we could achieve was only 0.214 (which is already likely to be enhanced by the presence of loop edges, i.e. countries reporting on themselves), which suggests that the network is organized around a dominant 'core' surrounded by nodes that connect to the core only via weak links. Assuming that important countries are well connected and globally centred (as supported by our cluster analysis), further analyses were conducted to identify these countries based on their centrality, using *k*-core decomposition [25].

The *k*-core analysis is a step by step process where the least connected nodes are gradually removed from the graph. The whole network is called the 0-core. Removing isolated nodes (having no links) result in the so-called 1-core. The process continues by removing the nodes having only a single link (thus their degree is 1). This may have created other nodes that are left with only a single link, so the process is repeated until there are no more nodes with degree 1. The remaining network is called the 2-core, since the degree of each node is at least 2. Removing all nodes having degree 2 yields the 3-core and so on. The process ends when we removed all nodes. The *coreness *or *shell index *of a node is the largest *k *for which the node is the member of the *k*-core but not the member of the *k+1*-core. In summary, this iterative process results in a series of subgraphs that gradually reveal the globally central region of the original network and stops when no more layers can be peeled off.

**Figure 2:**
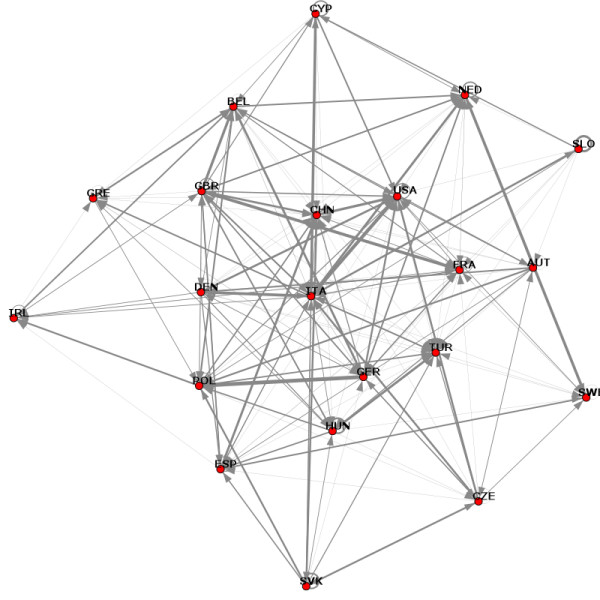
The innermost layer of the network obtained via *k*-core decomposition.

The *k-*core analysis revealed 12 layers (i.e. no countries could be eliminated so the remaining ones would have 13 connections). The innermost subgraph (the *12*-core) is depicted in Figure 2.

This *12*-core is the largest subgraph where each node has at least *12 *interconnections. The average edge weight within the subgraph is 7.35 ± 13.98 (max = 126.00). This subgraph consists of 21 countries with 190 connections, with 16 connections having a weight larger than mean + SD. These are: USA → USA (126), GER → TUR (73), ITA → ESP (68), ITA → CHN (58), GBR → GBR (51), GRE → CHN (45), GER → CHN (36), NED → USA (31), DEN → GER (30), ESP → ESP (28), GER → GER (27), ESP → CHN (27), ITA → TUR (27), DEN → FRA (26), GBR → CHN (23), FRA → TUR (22).

In practical terms, countries in this *12*-core subgraphs are the ones that have played an important role in food safety during the time period when the data were collected (January – November 2007, inclusive). However, this 'importance' may come from two very distinct sources: detector or transgressor which can be accounted for in the *k*-core decomposition. When considering this additional information, the 117 countries form two distinct 5-layer graphs. The transgressor 'layer 5' contains all countries on which at least 5 reports were made among each other whereas 'layer 0' shows countries on which no reports were made during the time period of this study. The detector 'layer 5' contains all countries who issued at least 5 reports on other countries in the same layer whereas 'layer 0' is formed by countries that made no reports (but intuitively, they are the ones that had been reported on, otherwise they would not have been included). Countries by layers are summarized in Table 1.

**Table 1:** Countries of the *k*-core layers obtained by in- (transgressor) and out- (detector) degree decompositions, respectively

**Layer^a^**	**In-coreness**		**Out-coreness**	
	**Countries**	**N**	**Countries**	**N**
Zero	LUX ISL	2	CHN THA IND TUR NGR IRI RSA INA EGY CHI UKR MAS PER MEX SYR RUS MYA ARG DOM KSA LIB HKG JPN PHI SUI COL KEN BRA GHA SMR TPE CRC VIE SRI ZIM TUN CIV PAR SIN BAN MAR PAK TAN GAM SEN SEY GUI PAN MDA SUR CRO KAZ HAI GEO ANG ALB MOZ ALG URU SLE ARM CAN MKD UGA NAM NCA UZB FIJ ISR BOL AUS OMA JOR ECU JAM MAW SCG ETH NZL MRI SUD GAB MAD BIH PRK MON	86
1^st^	EST MYA KSA MLT SMR ZIM CIV PAR ROM GUI MDA HAI ALB ALG SLE ARM UGA NAM UZB OMA JOR JAM MAW ETH NZL SUD MAD BIH PRK MON	30	LAT LUX POR ROM ISL	5
2^nd^	NOR FIN DOM LIB SLO KEN TPE SIN TAN GAM PAN SUR CRO GEO ANG MOZ URU FIJ ISR BOL SCG MRI GAB	23		0
3^rd^	NGR RSA CYP AUT LTU PER MEX JPN SEY KAZ MKD NCA ECU	13	EST POL BUL	3
4^th^	SVK CZE CHI DEN UKR HUN MAS LAT POR BUL SWE COL CRC TUN MAR SEN CAN AUS	18	USA ESP CYP SVK LTU HUN GRE FIN IRL MLT SWE	11
5^th^	USA CHN **GBR **THA ESP IND **FRA **TUR **ITA **IRI INA EGY **GER **POL **BEL **NED SYR GRE RUS ARG IRL HKG PHI SUI BRA GHA VIE SRI BAN PAK	30	**GBR FRA ITA **NOR CZE **GER **DEN AUT **BEL **NED SLO	11

^a ^layer 0 represent the most outside layer (the first layer to be 'peeled off') whereas layer 5 is the innermost layer where no further reduction can be made

**Figure 3:**
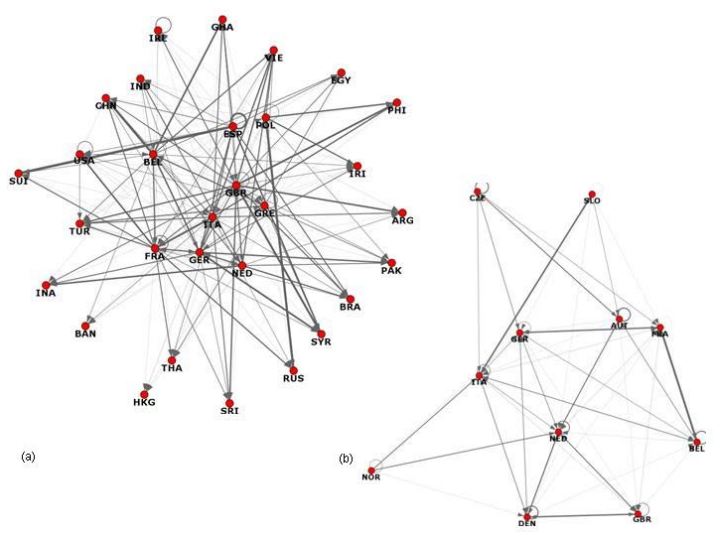
The innermost (5^th^) layer of the k-core decomposition based on in-degrees (a) and (b) out-degrees.

The 5^th ^layer for both detectors and transgressors are depicted separately in Fig 3, with a notable size difference. Only 11 European countries appear to play an important role in policing industrial food safety, mostly from the European Union (EU). Among the transgressors, only 36% were European and 30% from the EU.

The number of transgressors (of the 5^th ^layer) appeared to be independent of the population (hence remotely to the market size). Population statistics were obtained from the CIA's 2008 World Factbook [26]. The rank correlation between the number of reports received and population was small and non-significant (Kendall tau = .168, p = .2025).

Interestingly, there was a noticeable overlap between the transgressor and detector graphs for the UK, France, Italy, Germany and Belgium. These countries seem to guard 'food safety' and report all problems/hazards, regardless of the origin. Not surprisingly, three of these countries (UK, France and Germany) not only reported faulty food products from other countries but also made a considerable number of reports on their own products (Fig 1).

Owing to the noticeable difference between the set of transgressors and detectors, this aspect was further analyzed focusing exclusively on countries that are most involved in testing and those with a defective food product. All data are provided in Additional file 1: (Weighted and unweighted *in-degree *(transgressor) and *out-degree *(detector) for all countries).

### Breakdown of reporting patterns

**Figure 4:**
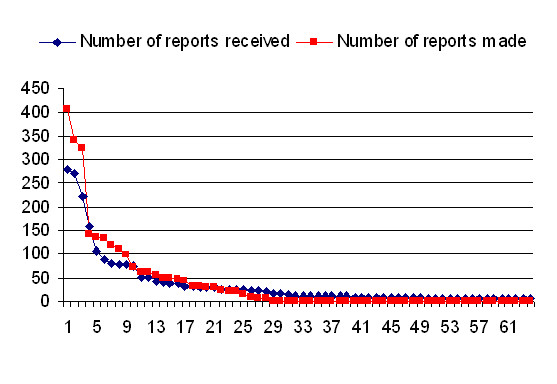
The pattern of reports (y axis) with reporting (*detector*) in red and reported (*transgressor*) in blue sorted by country (x axis).

An overview of reporting patterns demonstrates that the reporting behaviour is heavily skewed towards a small number of countries, both in terms of reporting and those being extensively reported (Table 1). Figure 4 shows that the number of reports logged or received by the 117 counties decreases rapidly. The highest number of reports made during the eleven-month period (406) exceeded the highest number of reports received (281) considerably.

Reports were made against products produced by the 115 countries, with the top 5 countries having received between 100 to 281 reports in the 11 month period. The next twenty countries received between 25 to 100 reports against their products in this period. The numbers further decline steadily from 23 to 1 report being made for products from the next 88 countries, with no reports against products from Israel and Luxembourg. Ten countries that are most frequent transgressors provided sixty per cent of all faulty products marketed.

In contrast, the pattern for the 30 detectors is that the top three make over 300 reports each (Italy 406, Germany 340, UK 322) as detailed in Additional file 1: (Weighted and unweighted *in-degree *(transgressor) and *out-degree *(detector) for all countries). The next five countries make between 99 and 142 reports with the final 20 countries making between 71 and 1 reports. In all, the top ten detectors account for three quarters of reports.

**Figure 5:**
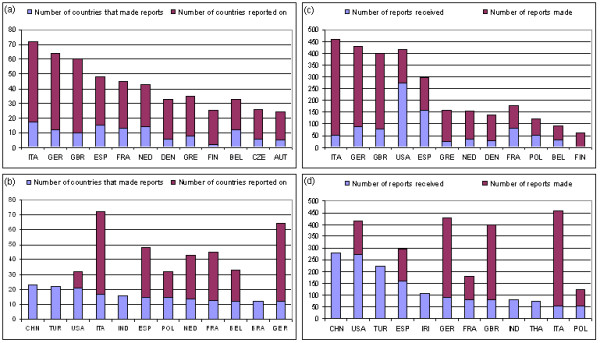
**Total trends in reporting for the top 12 countries with blue = 'transgressor' and red = 'detector':** a) Sorted by country making the highest number of reports against other countries; b) Sorted by number of other countries making reports against the selected country; c) Sorted by total number of reports made by against all products; d) Sorted by total number of reports against products from the selected country.

Twelve countries constitute the most active level with the reporting activities highlighted in Figure 5a for numbers of transgressors. Relationships are given for both detectors (in red) and transgressors (in blue). Considerable variations in reports occur when both the quantities and countries are examined (Fig 5). Large variations exist for the top twelve countries between those reporting and those reported. A lesser degree of variation was observed whether reports relate to countries or products (i.e. Fig 5a versus 5c).

### Detector Countries

Reporting rates varied enormously between countries with no clear relationship with population size. The twelve-country segment selected with optimal rates of reporting differs from those for reported activities which may reflect differences in market size. EU countries predominate with each of Italy, Germany and the UK reporting products from over 50 countries (Fig 5a). This pattern remains when the total number of reports is considered (Fig 5c). However, the pattern of detectors changes when total reports are considered as the USA enters at number 4 signifying it makes a lot of reports from against fewer countries. In fact the USA made 126 reports against its own products during the period which may reflect the market size.

### Transgressor Countries

As expected for this segment, countries with larger populations often predominate, reflecting market size. However, this generalization frequently breaks down when the data are analyzed for individual countries as detectors or as transgressors. The order of reports against a country's products is given in Figure 5b in terms of numbers of transgressors. Of the core twelve countries, four are present owing only to reports against their products (China, Turkey, India and Brazil) and they have no reports against products arising from other countries. In contrast, a relatively large number of products from other countries have been reported by Italy and Germany, with a significantly reduced number of countries reporting them. This trend is followed by four countries, albeit to a lesser degree (Spain, Netherlands, France and Belgium).

In terms of total numbers of reports against a country, China leads, closely followed by the USA and Turkey. The pattern continues with countries from the EU in the order: Spain, Ireland, Germany, France and the UK.

On consideration of the total number of reports, the pattern exhibited for numbers of transgressors is reflected in the data for total number of products reported (Figure 5b and 5d). The same four countries have no reports of products from other countries with China and Turkey having a large number of reports against their products (> 200). Italy and Germany remain at the top in terms of foreign product reporting but with few reports against their products by other countries. Similarly, there are a large number of reports against products from the USA, which has in turn reported many products.

### Limitations and extensions

The analyses were limited to approximately one year period. By expanding the timeframe of the analysis, trends and effects of various legislative and regulatory changes, such as the Beijing Declaration, can be investigated. As the reasons for food alerts/recalls vary greatly from illegal import (1.1%) through unauthorized food additives or ingredient (11.2%) to mycotoxin contamination (23.7%), future research would benefit from focused analyses. The methodology used for this paper can also be applied to various groups of countries based on the reasons for food alerts/recalls. Particular interests are reports owing to i) processing, ii) microbiological or iii) chemical contaminations.

## Conclusion

The vast majority of food reports are made by 10 countries out of the 117 studied, with EU countries predominating. The majority of the faulty foodstuffs originate in ten countries with four major food producers making no reports at all. A substantial worldwide improvement in food testing and reporting is required in response to the Beijing Declaration.

## Competing interests

The authors declare that they have no competing interests.

## Authors' contributions

TN conducted the network analyses, prepared the network visualisations and assisted in writing the manuscript. AP prepared the data for network analyses, conducted statistical analyses and assisted in writing the manuscript. DN suggested the study, sought the data, assisted with the data interpretation and contributed to writing the manuscript. All authors have read and approved the final version of the manuscript.

## Pre-publication history

The pre-publication history for this paper can be accessed here:



## Supplementary Material

Additional file 1 Weighted and unweighted *in-degree *(transgressor) and *out-degree *(detector) for all countries. The data show weighted and unweighted directional degrees for all 117 countries, in alphabetical order by country codes. Click here for file

Additional file 2 Definition of *modularity *and *k-core*. The file provides definitions for the two structural properties (modularity and k-core) used in this paper to describe the food recall pattern observed between January and November 2007. Click here for file

## References

[B1] World Health Organization. http://www.who.int/foodsafety/fs_management/meetings/forum07/en/index.html.

[B2] Kok EJ, Keijer J, Meter GA, Kuiper HA (2008). Comparative safety assessment of plant-derived foods. Regul Toxicol Pharmacol.

[B3] Hugas M, Tsigarida E, Robinson T, Calistri P (2007). Risk assessment of biological hazards in the European Union. Int J Food Microbiol.

[B4] Sergent T, Ribonnet L, Kolosova A, Garsou S, Schaut A, De Saeger S, Van Peteghem C, Larondelle Y, Pussemier L, Schneider Y-J (2008). Molecular and cellular effects of food contaminants and secondary plant components and their plausible interactions at the intestinal level. Food Chem Toxicol.

[B5] Birchard K (2001). Europe tackles consumers fears over food safety. Lancet.

[B6] Millstone E, Lang T, Naska A, Eames M, Barling D, van Zwanenberg P, Trichopoulou A (2000). 'European Policy on Food Safety': Comments and suggestions on the White Paper on Food Safety. Trends Food Sci Tech.

[B7] Bergeaud-Blackler F, Paola Ferretti M (2006). More politics, stronger consumers? A new division of responsibility for food in the European Union. Appetite.

[B8] Coppens P, Fernandes da Silva M, Pettman S (2006). European regulations on nutraceuticals, dietary supplements and functional foods: A framework based on safety. Toxicology.

[B9] Paulsen P, Luf W, Smulders FJM, Pico Y (2007). Different legislations on toxicants in foodstuffs. Food Toxicants Analysis.

[B10] Halkier B, Holm L (2006). hifting responsibilities for food safety in Europe: An introduction. Appetite.

[B11] Marvin HJP, Kleter GA, Prandini A, Dekkers S, Bolton DJ (2007). Early identification systems for emerging foodborne hazards. Food Chem Toxicol.

[B12] Rapid Alert System for Food and Feed (RASFF). http://ec.europa.eu/food/food/rapidalert/index_en.htm.

[B13] Kleter GA, Prandini A, Filippi L, Marvin HJP (2007). Identification of potentially emerging food safety issues by analysis of reports published by the European Community's Rapid Alert System for Food and Feed (RASFF) during a four-year period. Food Chem Toxicol.

[B14] R Development Core Team (2006). R: A language and environment for statistical computing.

[B15] Csárdi G, Nepusz T (2006). The igraph software package for complex network research. InterJournal Complex Systems.

[B16] Newman MEJ (2003). The structure and function of complex networks. SIAM Review.

[B17] Palumbo MC, Farina L, Colosimo A, Tun K, Dhar PK, Giuliani A (2006). Networks everywhere? Some general implications of an emergent metaphor. Current Bioinformatics.

[B18] Watts DJ, Strogatz SH (1998). Collective dynamics of "small world" networks. Nature.

[B19] Ma'ayan A, Jenkins SL, Goldfarb J, Iyengar R (2007). Network analysis of FDA approved drugs and their targets. Mt Sinai J Med.

[B20] Nacher JC, Schwartz JM (2008). A global view of drug-therapy interactions. BMC Pharmacol.

[B21] Prolx SR, Promislow DEL, Phillips PC (2005). Network thinking in ecology and evolution. Trends Ecol Evol.

[B22] Kleemann R, Verschuren L, van Erk MJ, Nikolsky Y, Cnubben NHP, Verheij ER, Smilde AK, Hendriks HFJ, Zadelaar S, Smith GJ, Kaznacheev V, Nikolskaya T, Melnikov A, Hurt-Camejo E, Greef J van der, van Ommen B, Kooistra T (2007). Atherosclerosis and liver inflammation induced by increased dietary cholesterol intake: a combined transcriptomics and metabolomics analysis. Genome Biol.

[B23] Newman MEJ (2004). Fast algorithm for detecting community structure in networks. Phys Rev E Stat Nonlin Soft Matter Phys.

[B24] Latapy M, Pons P (2006). Computing communities in large networks using random walks. J Graph Algorithms Appl.

[B25] Seidman SB (1983). Network structure and minimum degree. Soc Networks.

[B26] Central Intelligence Agency:2008 The World Factbook. ISSN 1553-8133. https://www.cia.gov/library/publications/the-world-factbook/index.html.

[B27] Newman MEJ, Girvan M (2004). Finding and evaluating community structure in networks. Phys Rev E Stat Nonlin Soft Matter Phys.

