# Supplementary material for: Worldwide food recall patterns over an eleven month period: A country perspective
Source: BMC Public Health. 2008 Sep 10;8:308. doi: 10.1186/1471-2458-8-308 (PMC2556336; doi:10.1186/1471-2458-8-308)
Supplement: Additional file 1 — Weighted and unweighted in-degree (transgressor) and out-degree (detector) for all countries. The data show weighted and unweighted directional degrees for all 117 countries, in alphabetical order by country codes. [file 1471-2458-8-308-S1.doc]

**Additional file 1: Weighted and unweighted *in-degree* (transgressor) and *out-degree* (detector) for all countries (n = 117) in alphabetical order by country codes**

| **Country code** | **Country** | **In-degree** | **Out-degree** | **Sum of weighted in-degree** | **Sum of weighted out-degree** |
| --- | --- | --- | --- | --- | --- |
| ALB | Albania | 1 | 0 | 1 | 0 |
| ALG | Algeria | 1 | 0 | 1 | 0 |
| ANG | Angola | 2 | 0 | 6 | 0 |
| ARG | Argentina | 11 | 0 | 31 | 0 |
| ARM | Armenia | 1 | 0 | 1 | 0 |
| AUS | Australia | 5 | 0 | 8 | 0 |
| AUT | Austria | 5 | 19 | 8 | 45 |
| BAN | Bangladesh | 5 | 0 | 13 | 0 |
| BEL | Belgium | 12 | 21 | 30 | 61 |
| BIH | Bosnia and Herzegovina | 1 | 0 | 1 | 0 |
| BOL | Bolivia | 2 | 0 | 2 | 0 |
| BRA | Brazil | 12 | 0 | 38 | 0 |
| BUL | Bulgaria | 4 | 8 | 5 | 9 |
| CAN | Canada | 6 | 0 | 6 | 0 |
| CHI | Chile | 9 | 0 | 11 | 0 |
| CHN | China | 23 | 0 | 281 | 0 |
| CIV | Cote d’Ivoire | 1 | 0 | 6 | 0 |
| COL | Colombia | 6 | 0 | 7 | 0 |
| CRC | Costa Rica | 5 | 0 | 7 | 0 |
| CRO | Croatia | 2 | 0 | 5 | 0 |
| CYP | Cyprus | 4 | 15 | 11 | 29 |
| CZE | Czech Republic | 6 | 20 | 25 | 55 |
| DEN | Denmark | 6 | 27 | 28 | 109 |
| DOM | Dominican Republic | 2 | 0 | 3 | 0 |
| ECU | Ecuador | 3 | 0 | 3 | 0 |
| EGY | Egypt | 10 | 0 | 28 | 0 |
| ESP | Spain | 15 | 33 | 160 | 136 |
| EST | Estonia | 1 | 9 | 1 | 14 |
| ETH | Ethiopia | 1 | 0 | 2 | 0 |
| FIJ | Fidji | 2 | 0 | 2 | 0 |
| FIN | Finland | 2 | 23 | 2 | 61 |
| FRA | France | 13 | 32 | 81 | 99 |
| GAB | Gabon | 2 | 0 | 2 | 0 |
| GAM | The Gambia | 2 | 0 | 3 | 0 |
| GBR | United Kingdom | 10 | 50 | 78 | 322 |
| GEO | Georgia | 2 | 0 | 3 | 0 |
| GER | Germany | 12 | 52 | 89 | 340 |
| GHA | Ghana | 7 | 0 | 26 | 0 |
| GRE | Greece | 8 | 27 | 25 | 132 |
| GUI | Guinea | 1 | 0 | 1 | 0 |
| HAI | Haiti | 1 | 0 | 1 | 0 |
| HKG | Hong Kong | 11 | 0 | 43 | 0 |
| HUN | Hungary | 8 | 12 | 12 | 24 |
| INA | Indonesia | 9 | 0 | 18 | 0 |
| IND | India | 16 | 0 | 78 | 0 |
| IRI | Iran | 12 | 0 | 106 | 0 |
| IRL | Ireland | 7 | 12 | 11 | 20 |
| ISL | Iceland | 0 | 1 | 0 | 1 |
| ISR | Israel | 2 | 0 | 4 | 0 |
| ITA | Italy | 17 | 55 | 52 | 406 |
| JAM | Jamaica | 1 | 0 | 2 | 0 |
| JOR | Jordan | 1 | 0 | 1 | 0 |
| JPN | Japan | 4 | 0 | 6 | 0 |
| KAZ | Kazakhstan | 3 | 0 | 5 | 0 |
| KEN | Kenya | 2 | 0 | 2 | 0 |
| KSA | Saudi Arabia | 2 | 0 | 4 | 0 |
| LAT | Latvia | 7 | 4 | 12 | 7 |
| LIB | Lebanon | 2 | 0 | 1 | 0 |
| LTU | Lithuania | 4 | 15 | 6 | 28 |
| LUX | Luxembourg | 0 | 3 | 0 | 5 |
| MAD | Madagascar | 1 | 0 | 1 | 0 |
| MAR | Morocco | 5 | 0 | 18 | 0 |
| MAS | Malaysia | 7 | 0 | 20 | 0 |
| MAW | Malawi | 1 | 0 | 2 | 0 |
| MDA | Moldova | 1 | 0 | 3 | 0 |
| MEX | Mexico | 3 | 0 | 6 | 0 |
| MKD | Macedonia | 3 | 0 | 5 | 0 |
| MLT | Malta | 1 | 9 | 3 | 32 |
| MON | Monaco | 1 | 0 | 1 | 0 |
| MOZ | Mozambique | 2 | 0 | 3 | 0 |
| MRI | Mauritius | 3 | 0 | 3 | 0 |
| MYA | Myanmar(Burma) | 1 | 0 | 2 | 0 |
| NAM | Namibia | 1 | 0 | 3 | 0 |
| NCA | Nicaragua | 4 | 0 | 9 | 0 |
| NED | Netherlands | 14 | 29 | 38 | 118 |
| NGR | Nigeria | 3 | 0 | 41 | 0 |
| NOR | Norway | 2 | 17 | 2 | 48 |
| NZL | New Zealand | 1 | 0 | 1 | 0 |
| OMA | Oman | 1 | 0 | 2 | 0 |
| PAK | Pakistan | 7 | 0 | 23 | 0 |
| PAN | Panama | 2 | 0 | 9 | 0 |
| PAR | Paraguay | 1 | 0 | 1 | 0 |
| PER | Peru | 4 | 0 | 10 | 0 |
| PHI | Philippines | 6 | 0 | 12 | 0 |
| POL | Poland | 15 | 17 | 52 | 71 |
| POR | Portugal | 6 | 9 | 8 | 19 |
| PRK | North Korea (PDR of Korea) | 1 | 0 | 1 | 0 |
| ROM | Romania | 1 | 1 | 1 | 1 |
| RSA | South Africa | 3 | 0 | 6 | 0 |
| RUS | Russia | 7 | 0 | 9 | 0 |
| SCG | Serbia and Montenegro | 2 | 0 | 4 | 0 |
| SEN | Senegal | 5 | 0 | 8 | 0 |
| SEY | Seychelles | 3 | 0 | 4 | 0 |
| SIN | Singapore | 2 | 0 | 5 | 0 |
| SLE | Sierra Leone | 1 | 0 | 2 | 0 |
| SLO | Slovenia | 2 | 17 | 3 | 42 |
| SMR | San Marino | 1 | 0 | 1 | 0 |
| SRI | Sri Lanka | 5 | 0 | 22 | 0 |
| SUD | Sudan | 1 | 0 | 1 | 0 |
| SUI | Switzerland | 6 | 0 | 7 | 0 |
| SUR | Suriname | 2 | 0 | 6 | 0 |
| SVK | Slovakia | 4 | 14 | 11 | 48 |
| SWE | Sweden | 6 | 12 | 7 | 31 |
| SYR | Syria | 7 | 0 | 9 | 0 |
| TAN | Tanzania | 2 | 0 | 5 | 0 |
| THA | Thailand | 11 | 0 | 75 | 0 |
| TPE | Taiwan | 3 | 0 | 3 | 0 |
| TUN | Tunisia | 4 | 0 | 12 | 0 |
| TUR | Turkey | 22 | 0 | 222 | 0 |
| UGA | Uganda | 1 | 0 | 2 | 0 |
| UKR | Ukraine | 7 | 0 | 26 | 0 |
| URU | Uruguay | 2 | 0 | 5 | 0 |
| USA | United States | 21 | 11 | 272 | 142 |
| UZB | Uzbekistan | 1 | 0 | 1 | 0 |
| VIE | Vietnam | 11 | 0 | 31 | 0 |
| ZIM | Zimbabwe | 1 | 0 | 1 | 0 |
