# Supplementary material for: Worldwide food recall patterns over an eleven month period: A country perspective
Source: BMC Public Health. 2008 Sep 10;8:308. doi: 10.1186/1471-2458-8-308 (PMC2556336; doi:10.1186/1471-2458-8-308)
Supplement: Additional file 2 — Definition of modularity and k-core. The file provides definitions for the two structural properties (modularity and k-core) used in this paper to describe the food recall pattern observed between January and November 2007. [file 1471-2458-8-308-S2.doc]

**Additional file 2: Definition of *modularity* and *k-core***

Modularity is a quality measure for assessing different node partitions of a network in terms of how appropriately it represents the underlying clustered structure of the network [27]. Intuitively, a good partition groups densely connected nodes together; in other words, it strives to maximise the intra-group edge (link) density while minimising the number of edges that go between groups. However, this intuitive description needs to be refined, since a trivial partition, which puts all the nodes in the same group definitely maximises the number of edges within groups, but it says nothing of interest about the structure of the network. Therefore, a more precise description of a partition we are looking for is one that possesses more intra-cluster edges than what we would observe if we randomized the network while keeping the degrees of the vertices.

Given a degree distribution (i.e. a sequence of integers *k_0_, k_1_, k_2_*, ... giving the number of nodes with zero, one, two (and so on) adjacent edges), one can calculate the probability of the existence of an edge between node *i* and *j* under the assumption that the network is completely random (but follows the given degree distribution). It can be shown that this probability is *d_i_ d_j_ / 2m*, where *d_i_* is the degree of node *i*, *d_j_* is the degree of node *j* and *m* is the total number of edges in the network. Let *A_ij_* be 1 if there is an edge between nodes *i* and *j* and let it be 0 otherwise. *A_ij_*- ( *d_i_ d_j_ / 2m* ) is therefore the difference between the observed and the expected edge count between nodes *i* and *j* (the observed edge count is either 0 or 1, the expected one equals the probability of the existence of an edge under the assumption that the network is completely random). The modularity measure is obtained by summing these differences over all intra-cluster vertex pairs and dividing it by *2m*. The division is required in order to enable the comparison of modularity for networks with different edge counts. Higher modularity scores indicate better partitions. The trivial partitions (all nodes belong to the same community or all nodes belong to separate communities) yield zero modularity. As a rule of thumb, Newman and Girvan stated that a modularity larger than 0.3 indicates that the partition being assessed appropriately describes the underlying clustered structure of the network [27]. The clustered structure of a network is therefore usually unveiled by applying heuristic procedures to maximise the modularity (since an exhaustive search in the space of all possible partitions is infeasible) and then examining the partition that achieved the maximal modularity. One such heuristic we used is described in the paper of Latapy and Pons [24].
